# Highly Reproducible, Vendor‐Agnostic, Motion‐Insensitive Liver PDFF Mapping at 0.55T, 1.5T, and 3T

**DOI:** 10.1002/mrm.70223

**Published:** 2025-12-12

**Authors:** Jiayi Tang, Daiki Tamada, Jon‐Fredrik Nielsen, Jitka Starekova, Julius F. Heidenreich, Felix Schön, Alexandra A. Anagnostopoulos, Amirhossein Roshanshad, Lu Mao, Shohei Fujita, Pengcheng Xu, Christopher Keen, Imam Ahmed Shaik, Eugene Milshteyn, Seonghwan Yee, Andrew J. Ellison, David Rutkowski, Jeff Kammerman, Jean H. Brittain, Xiaodong Zhong, William A. Grissom, Maxim Zaitsev, Aaron L. Carrel, Yogesh Rathi, Yun Jiang, Berkin Bilgic, Scott B. Reeder, Diego Hernando

**Affiliations:** ^1^ Medical Physics University of Wisconsin‐Madison Madison Wisconsin USA; ^2^ Department of Radiology University of Wisconsin‐Madison Madison Wisconsin USA; ^3^ Functional MRI Laboratory, Department of Radiology University of Michigan Ann Arbor Michigan USA; ^4^ Department of Diagnostic and Interventional Radiology University Hospital Würzburg Würzburg Germany; ^5^ Institute and Polyclinic for Diagnostic and Interventional Radiology Faculty of Medicine and University Hospital Carl Gustav Carus Dresden, Dresden University of Technology Dresden Germany; ^6^ Biostatistics and Medical Informatics University of Wisconsin‐Madison Madison Wisconsin USA; ^7^ Athinoula A. Martinos Center for Biomedical Imaging Massachusetts General Hospital Charlestown Massachusetts USA; ^8^ Radiology Harvard Medical School Boston Massachusetts USA; ^9^ State Key Laboratory of Extreme Photonics and Instrumentation, College of Optical Science and Engineering Zhejiang University Zhejiang China; ^10^ Biomedical Engineering University of Michigan Ann Arbor Michigan USA; ^11^ Brigham and Women's Hospital Harvard Medical School Boston Massachusetts USA; ^12^ GE HealthCare Boston Massachusetts USA; ^13^ Radiology Massachusetts General Hospital Boston Massachusetts USA; ^14^ Biomedical Imaging Center, Chobanian and Avedisian School of Medicine Boston University Boston Massachusetts USA; ^15^ Calimetrix, LLC Madison Wisconsin USA; ^16^ Radiological Sciences University of California, Los Angeles Los Angeles California USA; ^17^ Biomedical Engineering Case Western Reserve University Cleveland Ohio USA; ^18^ Division of Medical Physics, Department of Diagnostic and Interventional Radiology, University Medical Center Freiburg, Faculty of Medicine University of Freiburg Freiburg Germany; ^19^ Pediatrics University of Wisconsin‐Madison Madison Wisconsin USA; ^20^ Radiology University of Michigan Ann Arbor Michigan USA; ^21^ Biomedical Engineering University of Wisconsin‐Madison Madison Wisconsin USA; ^22^ Medicine University of Wisconsin‐Madison Madison Wisconsin USA; ^23^ Emergency Medicine University of Wisconsin‐Madison Madison Wisconsin USA

**Keywords:** cross‐vendor, fat quantification, liver, MASLD, PDFF, proton density fat fraction, Pulseq, vendor‐agnostic

## Abstract

**Purpose:**

To develop and validate a vendor‐agnostic, motion‐insensitive proton‐density fat‐fraction (PDFF) quantification method.

**Methods:**

Flip‐angle‐modulated (FAM) 2D chemical‐shift‐encoded (CSE) MRI for PDFF quantification was implemented in both the vendor‐agnostic platform Pulseq (“Pulseq‐FAM”) and one vendor‐specific platform (“GE‐specific FAM”). These implementations were distributed to four sites with twelve MR systems of three vendors (Siemens/GE/Philips) and field strengths (0.55T/1.5T/3T). A sequentially‐shipped 16‐vial phantom (PDFF = 0%–30%/T1_water_ = 200–1400 ms) underwent confounder‐corrected PDFF mapping with commercial 3D‐CSE methods and GE‐specific FAM as available on each system, and Pulseq‐FAM on every system. To assess bias, phantom PDFF measurements were compared to reference. Between‐system variance was evaluated with linear mixed‐effects modeling. Different volunteers were also imaged at each site to assess free‐breathing PDFF mapping feasibility.

A prospective single‐site volunteer study was also conducted. Adult patients and children were imaged with breath‐held 3D‐CSE and free‐breathing GE‐specific and Pulseq‐FAM. Radiologists evaluated images for overall quality and motion artifacts. To assess bias, Pulseq‐FAM PDFF measurements were compared to 3D‐CSE and GE‐specific FAM. Test–retest repeatability was assessed by re‐imaging after repositioning. Between‐field‐strength reproducibility was assessed at 1.5T and 3.0T.

**Results:**

In the multi‐center study, Pulseq‐FAM showed reduced T1‐bias and between‐system variability versus 3D‐CSE in phantom PDFF measurements, and free‐breathing feasibility in volunteers. In the single‐site volunteer study (*N* = 57), Pulseq‐FAM improved image quality and motion artifacts versus 3D‐CSE (*p* < 0.01). Pulseq‐FAM showed excellent agreement with 3D‐CSE (95% limits‐of‐agreement (LoA) = 3.4% PDFF) and GE‐specific FAM (LoA = 2.0%). Pulseq‐FAM showed excellent repeatability (repeatability coefficient (RC) = 1.6% PDFF) and between‐field‐strength reproducibility (reproducibility coefficient (RDC) = 2.4%) versus 3D‐CSE (RC = 2.7%/RDC = 3.4%; differences *p* < 0.05).

**Conclusion:**

Pulseq‐FAM enables accurate, reproducible, vendor‐agnostic, and motion‐insensitive PDFF quantification in adults and children.

## 1 Introduction

Metabolic dysfunction‐associated steatotic liver disease (MASLD) affects approximately 30%–38% of the world adult population [1, 2]. Left untreated, MASLD can lead to further complications, including metabolic dysfunction‐associated steatohepatitis (MASH), fibrosis, cirrhosis, liver failure and liver cancer [3, 4]. Currently, several pharmacological and surgical interventions are undergoing or have completed (e.g., resmetirom [5] and semaglutide [6]) large‐scale clinical trials for the treatment of MASLD and MASH [7, 8]. An accurate, precise, and noninvasive method for the diagnosis, grading, and longitudinal monitoring of MASLD, with high reproducibility across study sites, would be highly desirable in the design of these clinical trials. Additionally, as a chronic disease, MASLD patients may receive care at multiple clinical sites over their lifetimes, further motivating the need for reproducible diagnosis and longitudinal treatment monitoring [9]. Considering the widespread prevalence of MASLD, highly accessible and reproducible methods for the assessment of MASLD would also empower public health strategies for population‐level disease screening and surveillance [10, 11].

Excess liver fat deposition is the earliest and hallmark feature of MASLD [12, 13]. Chemical‐shift‐encoded (CSE) MRI‐based methods have been shown to enable accurate, precise, and noninvasive quantification of proton‐density fat‐fraction (PDFF) as a biomarker for fat deposition in MASLD [14, 15]. Importantly, PDFF has been recently demonstrated to be the single best biomarker for diagnosing not only MASLD, but also its more aggressive subtypes, MASH and fibrotic MASH [16]. For these reasons, CSE‐MRI‐based liver PDFF quantification has enormous potential for the diagnosis, grading, and longitudinal monitoring of MASLD.

Current commercially available, FDA‐approved CSE‐MRI methods for PDFF quantification are typically implemented as 3D‐encoded, Cartesian, steady‐state spoiled gradient echo sequences acquired with constant flip angles [17, 18, 19]. Such methods are motion‐sensitive and require breath‐holding to mitigate motion artifacts, which may be challenging or inaccessible for patient groups such as children and the infirm. Breath‐holding also complicates clinical workflows if repeated acquisitions are needed due to inadvertent respiration [20, 21]. Further, even in patients who are able to sustain a breath hold, minor motion artifacts may still be present in resulting images, which degrade the repeatability of conventional 3D CSE‐MRI methods [22]. Alternative approaches, such as compressed sensing techniques, have been proposed to reduce the length of required breath‐holds in 3D‐CSE MRI, with acquisition times of 5–10 s achieved in previous works [23, 24, 25]. Radial methods have also been suggested to address the motion‐sensitivity limitation of 3D‐CSE [17, 26, 27]. However, such methods present other challenges, such as computationally intense reconstruction and residual motion‐related artifacts.

A CSE‐MRI method based on a sequential 2D‐encoded, Cartesian acquisition with flip angle modulation (FAM) has been recently proposed as a motion‐insensitive, high‐SNR, computationally simple alternative for PDFF quantification [28]. Due to the sequential 2D acquisition, each slice has a short temporal footprint that effectively freezes respiratory motion, enabling artifact‐free, free‐breathing acquisitions. To compensate for the SNR decrease in 2D‐encoded compared to 3D‐encoded sequences, FAM efficiently uses the equilibrium magnetization in a centric acquisition to increase signal at the center of k‐space. Flip angles are then modulated over the course of the phase encoding lines, as the acquisition moves into the outer regions of k‐space, to optimally balance k‐space filtering, quantitative accuracy, and SNR. In this way, FAM leverages the motion‐insensitivity of 2D‐encoded acquisitions, while mitigating their drawbacks. This improves quantitative performance and accessibility, especially for patient groups who may be unable to hold their breath adequately.

Although accessibility across patient groups is one component of overall access, accessibility across clinical sites and MR system vendors remains a critical unmet need. FAM was originally implemented and demonstrated on the sequence programming platform of a single vendor (GE HealthCare), limiting access to this technique. This limitation may be addressed by using a vendor‐agnostic approach. The vendor‐agnostic platform Pulseq [29] has received considerable interest from the MR community and is now compatible with several major MR manufacturers, including Siemens Healthineers [29], GE HealthCare [30], and Philips [31, 32, 33, 34]. For these reasons, Pulseq may be a suitable platform for implementing FAM such that it can be acquired equivalently across MR systems of different vendors.

Therefore, the purpose of this work is to develop and validate a vendor‐agnostic, Pulseq‐based method for highly accessible, motion‐insensitive PDFF mapping that works equivalently across vendors, field strengths, and patient populations. Further, this work aims to assess the bias, repeatability, and between‐system reproducibility of the proposed method in phantoms, children, and adults.

## 2 Methods

This section describes the development of the proposed vendor‐agnostic (Pulseq) FAM and its comparison to conventional 3D CSE‐MRI methods and the previously proposed vendor‐specific FAM. For clarity, we refer to 3D‐encoded, commercially available, breath‐held CSE‐MRI methods as “3D‐CSE”; the existing GE‐specific implementation of 2D‐encoded, motion‐robust FAM CSE‐MRI as “GE‐specific FAM”; and the Pulseq implementation of FAM as “Pulseq‐FAM”. As described below, GE‐specific FAM and Pulseq‐FAM share many acquisition parameters and identical flip angles; the main difference is the programming framework (vendor‐specific/proprietary vs. open‐source and vendor‐agnostic).

In this work, we evaluated the bias, between‐vendor and between‐center reproducibility, and noise performance of Pulseq‐FAM through a multi‐center phantom study. We also evaluated the in vivo free‐breathing feasibility of Pulseq‐FAM with multiple sites and vendors. Further, we performed a single‐site, single‐vendor prospective in vivo study to evaluate the image quality, bias, repeatability, and between‐field strength reproducibility of Pulseq‐FAM in patients.

### 2.1 Vendor‐Agnostic Pulseq Implementation

FAM was implemented in the Pulseq platform and distributed to four sites with a total of 12 MR systems, at 0.55T, 1.5T, and 3T, from three major vendors (Siemens Healthineers, GE HealthCare, and Philips; see Table 1). A Pulseq interpreter was installed on each system [29, 30, 33]. For each field strength, sequence parameters were modified to be within the capabilities of all systems used in this study. Monopolar readouts (flyback gradients) were used, and echo times were designed to have favorable noise performance in magnitude‐based PDFF mapping at each field strength [35]. Other imaging parameters for Pulseq‐FAM, as well as 3D‐CSE and GE‐specific FAM acquired for comparison, are given in Table 2. Flip angle schedules in GE‐specific FAM and Pulseq‐FAM were identical and optimized, based on previously published numerical optimization [22, 28], for imaging parameters at each field strength (see Figure S1). Parameters for 3D‐CSE were taken from clinical protocols in use at study sites.

**TABLE 1:** MR systems used in this work and available CSE‐MRI methods on each system.

Site	Vendor	Model	B_0_		Gradient strength	Gradient slew rate	Software version	3D‐CSE available	GE‐Specific FAM available	Pulseq‐FAM available
3	Siemens	Free.Max	0.55T		25 mT/m	40 T/m/s	XA60A			✓
1	GE	Artist	1.5T		33 mT/m	120 T/m/s	MR30.1	✓	✓	✓
1	Siemens	Avanto		45 mT/m	200 T/m/s	VE11C	✓		✓
3	Siemens	Sola		45 mT/m	200 T/m/s	XA61A	✓		✓
4	GE	Artist		33 mT/m	120 T/m/s	MR30.1	✓	✓	✓
1	Siemens	Skyra	2.89T	Collectively, “3T”	45 mT/m	200 T/m/s	XA30A			✓
3	Siemens	Vida			60 mT/m	200 T/m/s	XA60A	✓		✓
1	GE	Premier	3.0T		80 mT/m	200 T/m/s	MR30.1	✓	✓	✓
2	Philips	MR7700			65 mT/m	220 T/m/s	R5.9			✓
3	GE	Ultra‐High Performance (UHP)			100 mT/m	200 T/m/s	MR30.1	✓	✓	✓
3	GE	MR750			50 mT/m	200 T/m/s	MR30.1	✓	✓	✓
4	GE	Premier			80 mT/m	200 T/m/s	MR30.1	✓	✓	✓

**TABLE 2:** Acquisition parameters for CSE‐MRI methods used in this work.

Pulse sequence parameter	0.55T	1.5T				3T			
	Pulseq‐ FAM (2D)	3D‐CSE (IDEAL‐ IQ, GE)	3D‐CSE (LiverLab, Siemens)	GE‐Specific FAM (2D)	Pulseq‐ FAM (2D)	3D‐CSE (IDEAL‐ IQ, GE)	3D‐CSE (LiverLab, Siemens)	GE‐Specific FAM (2D)	Pulseq‐ FAM (2D)
TE_1_, first echo time (ms)	2.38	1.17	2.38	1.40	1.67	0.97	1.05	1.12	1.21
∆TE, echo time spacing (ms)	2.22	1.99	2.38	1.78	1.82	0.77	1.23	1.26	1.22
Number of echo trains × echoes per train	1 × 6	1 × 6	1 × 6	1 × 6	1 × 6	2 × 3	1 × 6	1 × 6	1 × 6
TR, repetition time (ms)	15.8	12.9	15.6	12.6	12.6	6.4	15.6	9.3	9.3
Total bandwidth (kHz)	125	125	172.8	125	125	250	172.8	250	250
Flip angle (°)	Variable	5	4	Variable	Variable	3	3	Variable	Variable
Field of view (cm × cm)	39 × 39	40 × 32	45 × 39.4	44 × 44	44 × 44	40 × 32	45 × 39.4	44 × 44	44 × 44
Slice thickness (mm)	8.0	8.0	7.0	8.0	8.0	8.0	7.0	8.0	8.0
Acquisition matrix	108 × 108	160 × 115	160 × 111	144 × 144	144 × 144	192 × 128	160 × 111	144 × 144	144 × 144
Typical number of slices in vivo	32	32	32	32	32	32	32	32	32
Parallel imaging acceleration (method, phase encode × slice encode nominal acceleration)	None	ARC [36], 2 × 2	CAIPIRINHA [37], 2 × 2	None	None	ARC [36], 2 × 2	CAIPIRINHA [37], 2 × 2	None	None
Temporal footprint per slice in vivo (s)	1.7	19	23	1.8	1.8	19	22	1.3	1.3
Typical total acquisition time in vivo (s)	54.4	19	23	57.6	57.6	19	22	41.6	41.6
Breathing state in vivo	Free‐breathing	End‐expiration breath hold	End‐expiration breath hold	Free‐breathing	Free‐breathing	End‐expiration breath hold	End‐expiration breath hold	Free‐breathing	Free‐breathing

*Note*: Parameters were shared between in vivo and phantom acquisitions. Parameters may have varied slightly for GE‐specific FAM in the single‐site in vivo study due to subject‐specific optimizations. Temporal footprint per slice refers to the duration required in an acquisition to collect data that contributes towards one slice's reconstruction. This is equal to the total acquisition time in a 3D‐encoded acquisition, but sequential 2D‐encoded acquisition (e.g., as in FAM) enables short per‐slice temporal footprint.

Some methods and results reported below were first reported in scientific abstracts [38, 39]. Further, some analysis methods below were also used in previous work [22].

### 2.2 Multi‐Center Phantom Study

We performed phantom experiments to evaluate the bias, reproducibility, and noise performance of Pulseq‐FAM.

In addition to motion‐sensitivity, a known technical limitation of steady‐state 3D‐CSE is that T1‐dependent differences in the steady‐state signal level of fat and water may lead to T1‐related bias in PDFF quantification [40, 41]. Indeed, conventional PDFF mapping based on steady‐state, low flip angle acquisitions, fundamentally presents a tradeoff between residual T1 bias and SNR. In contrast, FAM acquires each slice with centric encoding of magnetization not in steady‐state. This strategy inherently avoids T1‐related bias near the center of k‐space because all species, regardless of T1, start from equilibrium magnetization. To maintain quantitative accuracy, T1 effects at the outer regions of k‐space are considered as part of the optimization formulation that determines the modulated flip angles in FAM. The optimization formulation also considers SNR and k‐space filtering to achieve high overall image quality.

To enable assessment of the effect of T1 on the CSE‐MRI methods evaluated in this work, we imaged a PDFF‐T1 phantom at multiple sites, as described below.

#### 2.2.1 Description of Phantom

We used a commercially available PDFF‐T1 phantom (available by special order, Calimetrix, Madison, WI). The phantom contains a 2D grid of 16 cylindrical vials containing gels with simultaneously controlled combinations of PDFF (0%, 10%, 20%, and 30%) and T1_water_ (T1_w_) (200, 600, 1000, and 1400 ms, with approximately equal T1_w_ between 1.5T and 3T [42, 43]). These values cover liver PDFF and T1_w_ ranges commonly observed in vivo, including T1_w_ values observed pre‐ and post‐contrast injection [44, 45].

#### 2.2.2 MRI Acquisition

The phantom was shipped sequentially to each site in the study. At each site, the phantom was imaged (as available on each system, see Table 1) with 3D‐CSE (IDEAL‐IQ, GE HealthCare, Waukesha, WI; LiverLab, Siemens Healthineers, Erlangen, Germany), GE‐specific FAM, and Pulseq‐FAM. Multichannel head coils were used on each system except the Site 3 GE MR750, where a DuoFLEX coil (MR Instruments, Minnetonka, MN) was used due to equipment availability. The phantom was positioned to have the long axis of its vials aligned with the bore of the MR system. Each CSE‐MRI method was then acquired in the axial plane for 10 repetitions without repositioning, to enable voxel‐wise noise characterization.

#### 2.2.3 MRI Reconstruction and Analysis

For PDFF fitting in all CSE‐MRI acquisitions, no vendor algorithms were used. All acquisitions were analyzed using custom, offline magnitude‐based fitting models [35, 46], which included temperature correction for phantoms [47] (shift of 0.11 ppm in the fat spectrum relative to water). Thus, the model closely approximated the actual phantom signal. This approach helped isolate other sources of variability, such as T1‐related bias, from the fitting model. All reconstructions were performed centrally by the coordinating study site. For 3D‐CSE, PDFF fitting was performed on vendor‐reconstructed echo images. For Pulseq‐FAM, we used vendor‐specific toolkits to load raw k‐space data, then performed a 2D Fourier transform, adaptive coil combination [48], and PDFF fitting identically across systems. For GE‐specific FAM, we used both the vendor echo image pipeline (as for 3D‐CSE) and the raw k‐space pipeline (as for Pulseq‐FAM) to verify the quantitative equivalence of these two processing pipelines. To conduct this verification, we selected a 1.5T system and a 3.0T system to compare summary PDFF values (see below) with linear regression for GE‐specific FAM processed from vendor echo images and from raw k‐space.

Voxel‐wise standard deviation (SD) across the 10 repeated acquisitions were computed from PDFF maps as a surrogate for noise.

Circular regions of interest (ROIs) with diameter 1.4 cm were placed at the center of each phantom vial, using the water‐only map generated as part of PDFF fitting for best visualization of the vials, and also to blind the analyst to the computed PDFF values. ROIs were then copied to the PDFF and voxel‐wise PDFF SD maps. Summary values for each ROI were computed by averaging voxels within the ROI on the maps, using medical image viewer software (Horos v3.3.6, Nimble LLC, Annapolis, MD). Reference PDFF values for each group of phantom vials with a particular nominal PDFF were obtained from the vial with T1_w_ = 200 ms, using a 3D‐CSE acquisition with 1° flip angle. Vials for each nominal PDFF were filled with the same batch of lipid emulsion, so they all share the same PDFF by construction. The use of the T1_w_ = 200 ms vial most closely matches the T1_fat_ (˜300 ms) of the phantom [43]. Combined with a low flip angle acquisition, this reduces T1‐related bias in our PDFF reference values. Bias was evaluated by comparing summary PDFF means to reference.

To compare the between‐platform reproducibility of 3D‐CSE and Pulseq‐FAM, summary PDFF values from the 10 repeated acquisitions, across all systems where both methods were available, were fitted with a linear mixed‐effects model. Reference PDFF, nominal T1_w_, and field strength (categorical, 1.5T or 3T), as well as interaction terms, were included as fixed effects. MR system and phantom vial were included as random effects to decompose variability into between‐system, between‐vial, and within‐cell (residual) components. We fit the model separately for 3D‐CSE and Pulseq‐FAM on the subset of systems where both methods were available to enable direct comparison of fixed effect coefficients and variance components. Differences in fitted parameters between methods were assessed with a random permutation test (100 000 samples) that re‐labels the method within matched system × vial cells to preserve the correlation structure. Linear mixed‐effects modeling was performed in Matlab 2024a (MathWorks, Natick, MA). All other statistical tests in this work were performed with Pingouin v0.5.5 [49]. The threshold for statistical significance was set at *p* = 0.05.

Noise performance was evaluated by comparing summary values for voxel‐wise PDFF SD values, normalizing for each system by the median ROI for 3D‐CSE, to account for hardware (e.g., coil) differences between systems. Statistical significance of differences in normalized SD values was evaluated with a two‐tailed, two‐sample *t*‐test with Welch correction for unequal variances.

### 2.3 Multi‐Center In Vivo Feasibility Study

In addition to imaging the phantom on each system, several sites imaged healthy volunteers with informed written consent and under local Institutional Review Board (IRB) approval, to establish feasibility of Pulseq‐FAM for free‐breathing PDFF mapping in various systems. The volunteers differed between sites. A flexible abdominal coil was used, again except at the Site 3 GE MR750, where the same DuoFLEX coil was used as above due to equipment availability. PDFF maps were reconstructed offline using a graph‐cut algorithm [35] and hybrid fitting [46, 50] (a weighted combination of magnitude and complex‐based fitting), to mimic a common 3D‐CSE implementation as it would be used in a clinical context. The resultant PDFF maps were examined for apparent image quality and obvious artifacts.

### 2.4 Single‐Site In Vivo Image Quality and Quantitative Study

We evaluated the in vivo performance of Pulseq‐FAM compared to 3D‐CSE and GE‐specific FAM, in terms of image quality, bias, test–retest repeatability, and between‐field strength reproducibility. To this end, we conducted a prospective study at a single site in adult and children volunteers, with informed written consent and IRB approval.

#### 2.4.1 Description of Subjects

We recruited adult patients with suspected liver steatosis and/or iron overload, to enable assessment of our CSE‐MRI methods in a cohort with a diverse range of PDFF, as well as a wide range of R2*, which is known to confound PDFF quantification. We also recruited children with normal (5–85th percentile) and elevated (> 95th percentile) body mass index (BMI), with the aim to assess performance in children with a diverse range of PDFF, given the known correlation between BMI and PDFF [51, 52, 53]. A subset of adult subjects (depending on subjects' availability) were imaged at both 1.5T (Signa Artist, GE HealthCare) and 3.0T (Signa Premier, GE HealthCare) during the same study visit to assess reproducibility across field strengths. All other adults were imaged at 1.5T only (Signa Artist). Children were imaged at 3.0T only (Signa Premier). All acquisitions were performed using a large abdominal flexible coil (AIR, GE HealthCare) in addition to the spine coil built into the system table.

#### 2.4.2 MRI Acquisition

All CSE‐MRI methods were acquired in the axial plane. 3D‐CSE (IDEAL‐IQ, GE HealthCare) was acquired during breath‐holding at end‐expiration. GE‐specific FAM and Pulseq‐FAM were acquired during free‐breathing. To evaluate test–retest repeatability, all acquisitions were repeated after removing subjects from the bore, repositioning the subjects, and reacquiring localizers.

#### 2.4.3 MRI Reconstruction

PDFF maps were reconstructed using the same processing workflows as in the multi‐center phantom study, but with hybrid magnitude/complex fitting instead of magnitude fitting [46, 50], to mimic a clinical 3D‐CSE implementation. Within an exam, no acquisitions were repeated due to motion artifacts, to avoid biased image quality ratings in the reader study (see below).

Note that during this single‐site in vivo study, an update in the Pulseq interpreter for GE systems [30] modified RF scaling behavior. (The study site updated from pre‐release commit b4fd15e to release v1.9.0.) As noted in previous work [22], FAM is expected to be quantitatively robust to B1+ inhomogeneity, which is mimicked by changes to RF scaling. However, qualitative changes in image appearance may result. Therefore, we use post‐update volunteer data in the reader study below, and pre‐ and post‐update data for quantitative analysis. We provide further comparisons of pre‐ and post‐update data in the supplemental materials.

#### 2.4.4 Reader Study

Following an approach reported in previous work [21, 22], PDFF maps from test acquisitions (i.e., before patient repositioning) were evaluated by three radiologists (J.S., J.F.H., F.S.) with 4–13 years of experience in abdominal MRI. Images were presented in different randomized orders to each reader, and readers were blinded to the acquisition method. A five‐point Likert scale was used to evaluate overall image quality and motion artifacts (5 = excellent quality, no artifacts; 4 = very good quality, minor artifacts; 3 = moderate quality, moderate artifacts; 2 = fair but still diagnostic quality, major artifacts; 1 = non‐diagnostic quality, severe artifacts). Inter‐reader reliability between the three readers was assessed using intraclass correlation coefficient with a two‐way random effects model, including averaged measures and absolute agreement (ICC(2,k)). For paired comparisons between CSE‐MRI techniques, ratings were pooled between all readers and two‐tailed Wilcoxon signed‐rank tests were calculated.

#### 2.4.5 Quantitative Analysis

Also following a standardized approach established in previous work [54], for each acquisition, two trained analysts (A.A.A., A.R.) placed one ROI within each of the nine Couinaud liver segments on all reconstructed PDFF maps. All analyses were conducted with medical image viewer software (Horos v3.3.6, Nimble LLC) under the supervision of a board‐certified radiologist (J.S.) with 13 years of experience in abdominal MRI. ROIs were elliptical, 2.8 ± 1.2 cm^2^ (mean ± SD) in area, and avoided bile ducts, large blood vessels, and obvious artifacts where possible. As performed in the phantom, summary PDFF values for each liver segment were computed by averaging PDFF values within ROIs. A whole‐liver PDFF measurement was then computed as the unweighted average of summary values of all nine liver segments [54]. Confounder‐corrected R2* maps were also generated as part of the process of correcting for R2* in PDFF quantification. ROIs with summary R2* values greater than 276 s^−1^ at 1.5T and 397 s^−1^ at 3.0T, according to the 3D‐CSE method, were excluded from analysis, based on modeling [55] that suggests the echo times used in Pulseq‐FAM limit its PDFF accuracy above these thresholds due to R2* decay.

The bias of PDFF measurements was evaluated by comparing whole‐liver PDFF measurements between Pulseq‐FAM and 3D‐CSE and GE‐specific FAM in linear regression and Bland–Altman analysis. The 95% limits of agreement (LoA) between two CSE‐MRI methods were calculated as 1.96 times the SD of all differences for whole‐liver PDFF measurements.

To evaluate repeatability, whole‐liver PDFF measurements were compared between test and retest acquisitions using Bland–Altman analysis. The repeatability coefficient (RC) was calculated as 1.96 times the SD of all test–retest value differences. Significance of differences in RC was tested by comparing the variances of test–retest differences with a two‐tailed Levene's test, with significance level set at *p* = 0.05.

To evaluate between‐field strength reproducibility at 1.5T and 3.0T, whole‐liver PDFF measurements were compared in the adult patients who were imaged at both field strengths, using Bland–Altman analysis. The reproducibility coefficient (RDC) was calculated as 1.96 times the SD of all whole‐liver PDFF differences between 1.5T and 3.0T. Significance of differences in RDC was again tested with a two‐tailed Levene's test.

## 3 Results

### 3.1 Phantom Study

**FIGURE 1:**
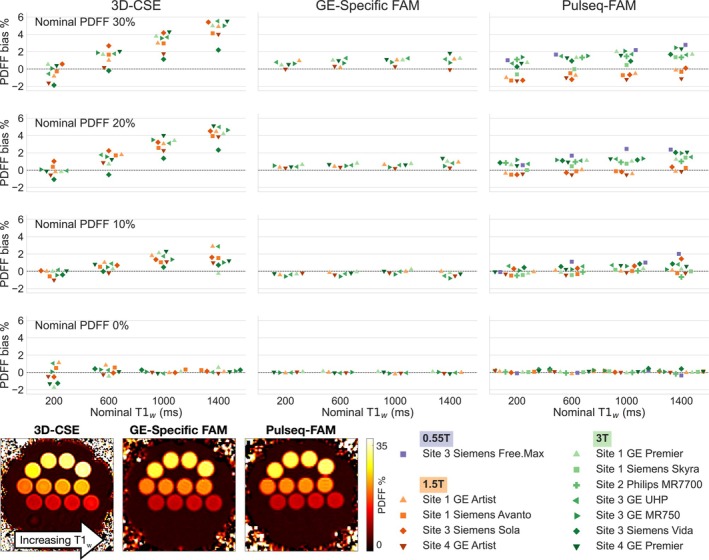
Pulseq‐FAM shows low bias in PDFF quantification across MR systems, field strengths, and vendors. A phantom with vials varying in PDFF and T1_water_ (T1_w_) was imaged with 3D‐CSE, GE‐specific FAM, and Pulseq‐FAM, as available on each system, and representative PDFF maps are shown. ROIs were drawn on each phantom vial, and the plot shows bias for summary values in each ROI. The vendors' 3D‐CSE methods show T1‐related bias as T1_w_ increases, as expected given the 3°–5° flip angles used. GE‐specific FAM and Pulseq‐FAM show good accuracy and virtually no T1‐bias across a wide range of T1_w_, as expected via the centric‐encoded acquisition. Note that within one nominal T1_w_ column in the plot, the points may be shifted slightly laterally to avoid overlapping for display only.

Figure S2 shows sample PDFF maps of the phantom, acquired with Pulseq‐FAM on each of the 12 systems in the study. Figure 1 illustrates that Pulseq‐FAM generally showed low bias of ≤ 3% PDFF, with reduced T1‐related bias compared to 3D‐CSE, although it showed slightly more bias than GE‐specific FAM. Linear regression between summary PDFF values obtained through the vendor echo image and raw k‐space processing pipelines for GE‐specific FAM showed strong agreement, with a slope with 95% confidence interval (CI) of (0.996, 1.005), intercept with CI (−0.15, 0.03), and R^2^ = 0.9998.

Table 3 shows results of linear mixed‐effects model fitting for 3D‐CSE and Pulseq‐FAM. As already noted in Figure 1, Pulseq‐FAM shows a significantly smaller coefficient for the reference PDFF × nominal T1_w_ interaction term compared to 3D‐CSE (0.02 vs. 0.16% PDFF per% PDFF·s; *p* < 0.01). For Pulseq‐FAM, the PDFF × T1_w_ slope increased modestly at 3T relative to 1.5T (three‐way interaction 0.02 [95% CI 0.01–0.02]). Pulseq‐FAM also shows a significantly higher coefficient for the reference PDFF × field strength 3T term compared to 3D‐CSE. This can also be seen in Figure 1, where 1.5T summary values for Pulseq‐FAM at higher values of nominal PDFF are slightly but consistently lower than 3T values. In addition, variance components demonstrate substantially improved reproducibility for Pulseq‐FAM: between‐system variance 0.042 versus 0.216 (−81%), between‐vial variance 0.015 versus 0.142 (−89%), and residual variance 0.074 versus 0.221 (−67%); all differences *p* < 0.01. Summed variance (system + vial + residual) was reduced to 0.131 from 0.579 (−77%), indicating tighter agreement across systems and vials after accounting for the fixed effects. These findings are also reflected in the lower and more consistent biases for Pulseq‐FAM in Figure 1.

**TABLE 3:** Linear mixed‐effects models for between‐system reproducibility, with fixed effect coefficients (β; 95% CI) and variance components with 95% CIs, fitted separately for 3D‐CSE and Pulseq‐FAM.

Parameter		3D‐CSE	Pulseq‐FAM	Significance of difference in parameter between 3D‐CSE and Pulseq‐FAM
Fixed effects coefficients	Intercept	0.14 (−0.64, 0.93)	0.20 (−0.09, 0.49)	
	Reference PDFF	0.96** (0.92, 0.99)	0.96** (0.95, 0.97)	
	Field strength 3T	−0.14 (−0.77, 0.49)	−0.18 (−0.46, 0.11)	
	Nominal T1_w_ (% PDFF/second of T1_w_)	0.01 (−0.69, 0.70)	0.00 (−0.23, 0.24)	
	Reference PDFF × field strength 3T	0.00 (−0.01, 0.01)	0.05** (0.05, 0.06)	**
	Reference PDFF × nominal T1_w_	0.16** (0.12, 0.19)	0.02** (0.01, 0.03)	**
	Field strength 3T × nominal T1_w_	0.19* (0.01, 0.37)	−0.02 (−0.12, 0.09)	
	Reference PDFF × field strength 3T × nominal T1_w_	0.00 (−0.01, 0.01)	0.02** (0.01, 0.02)	
Random effects variances	MR system	0.216 (0.085, 0.550)	0.042 (0.016, 0.107)	**
	Phantom vial	0.142 (0.069, 0.291)	0.015 (0.007, 0.031)	**
	Residual variance	0.221 (0.205, 0.238)	0.074 (0.069, 0.080)	**

*Note*: * indicates *p* < 0.05 and ** indicates *p* < 0.01 for significance of coefficients within a method and for difference between methods in the rightmost column.

As shown in Figure S3, GE‐specific FAM and Pulseq‐FAM show more noise in PDFF quantification compared to 3D‐CSE acquired on the same systems, with a greater difference at 1.5T compared to 3T (*p* < 0.01 for comparisons at each field strength between 3D‐CSE and Pulseq‐FAM, and 3D‐CSE and GE‐specific FAM). Pulseq‐FAM shows similar noise performance as GE‐specific FAM (*p* = 0.08 and 0.06 at 1.5T and 3T, respectively).

### 3.2 Multi‐Center In Vivo Feasibility Study

**FIGURE 2:**
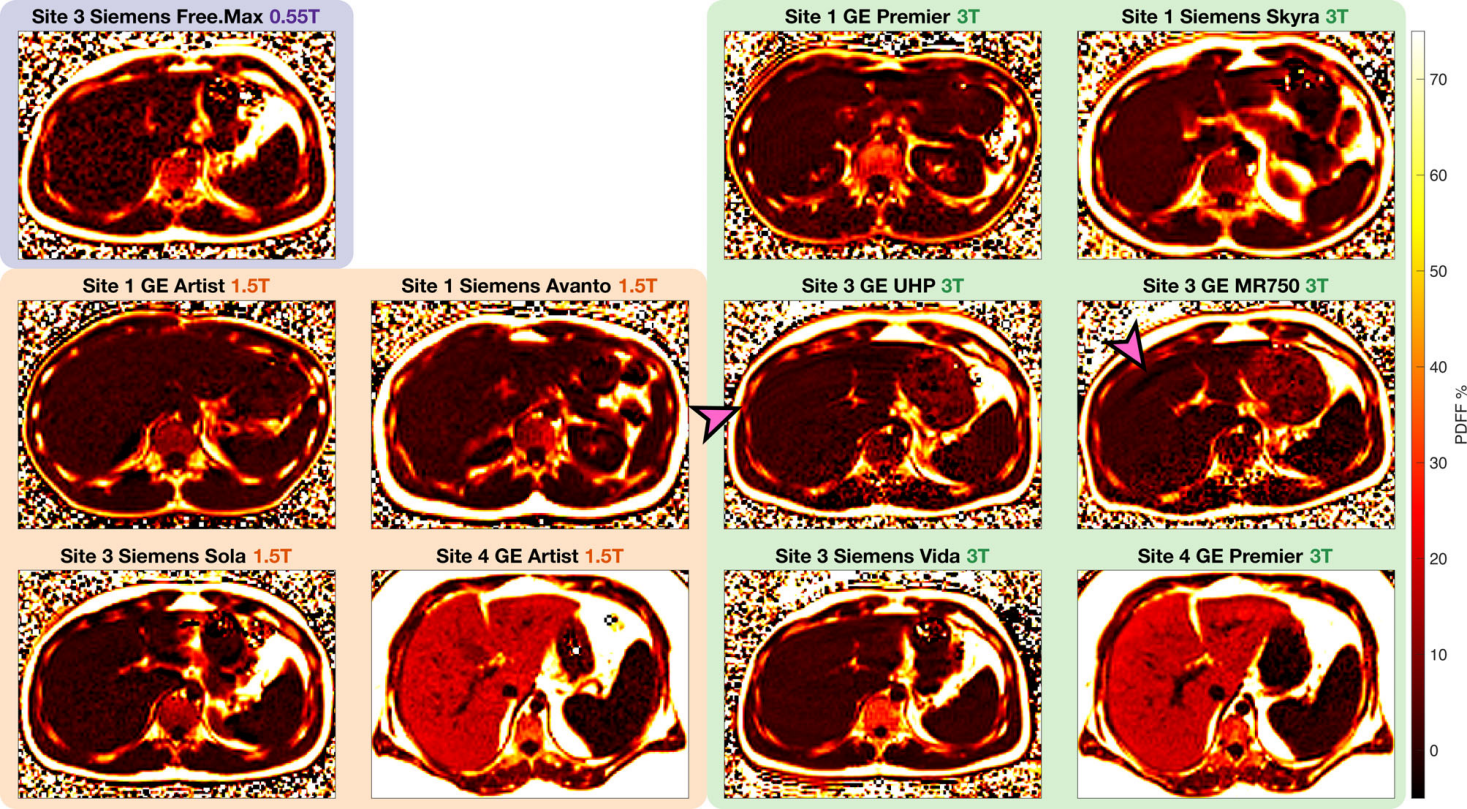
Pulseq‐FAM enables free‐breathing in vivo liver PDFF mapping with high image quality, regardless of vendor, MR system, and field strength. The figure shows example free‐breathing liver PDFF maps acquired with Pulseq‐FAM, of different volunteers with varying levels of liver fat, for each MR system in this study where in vivo imaging was possible. Pulseq‐FAM demonstrates free‐breathing in vivo feasibility with high apparent image quality in all hardware and site settings, although minor artifacts are visible on some 3T systems (arrows), and the 0.55T system shows lower apparent SNR.

Figure 2 shows sample Pulseq‐FAM PDFF maps from free‐breathing in vivo acquisitions on various MR systems. The images are of high apparent image quality and free of major motion or other artifacts, although minor artifacts can be seen on some systems. The lower apparent SNR of the 0.55T acquisition is also appreciable.

### 3.3 Single‐Site In Vivo Image Quality and Quantitative Study

#### 3.3.1 Cohort Characteristics

A total of 33 adults (ages 18–84, 55% male/45% female) were recruited and imaged. From these adult subjects, 20 had suspected liver steatosis and 13 had suspected iron overload. Of these, 11 adults with suspected steatosis and 10 adults with suspected iron overload were imaged at both 1.5T and 3.0T. Additionally, a total of 24 children (ages 10–14, 50% male/50% female) were also recruited, of which 12 children had normal (28–77th percentile) BMI and 12 had elevated (> 95th percentile) BMI. Overall, 14 adults and 9 children were imaged with the updated Pulseq interpreter.

**FIGURE 3:**
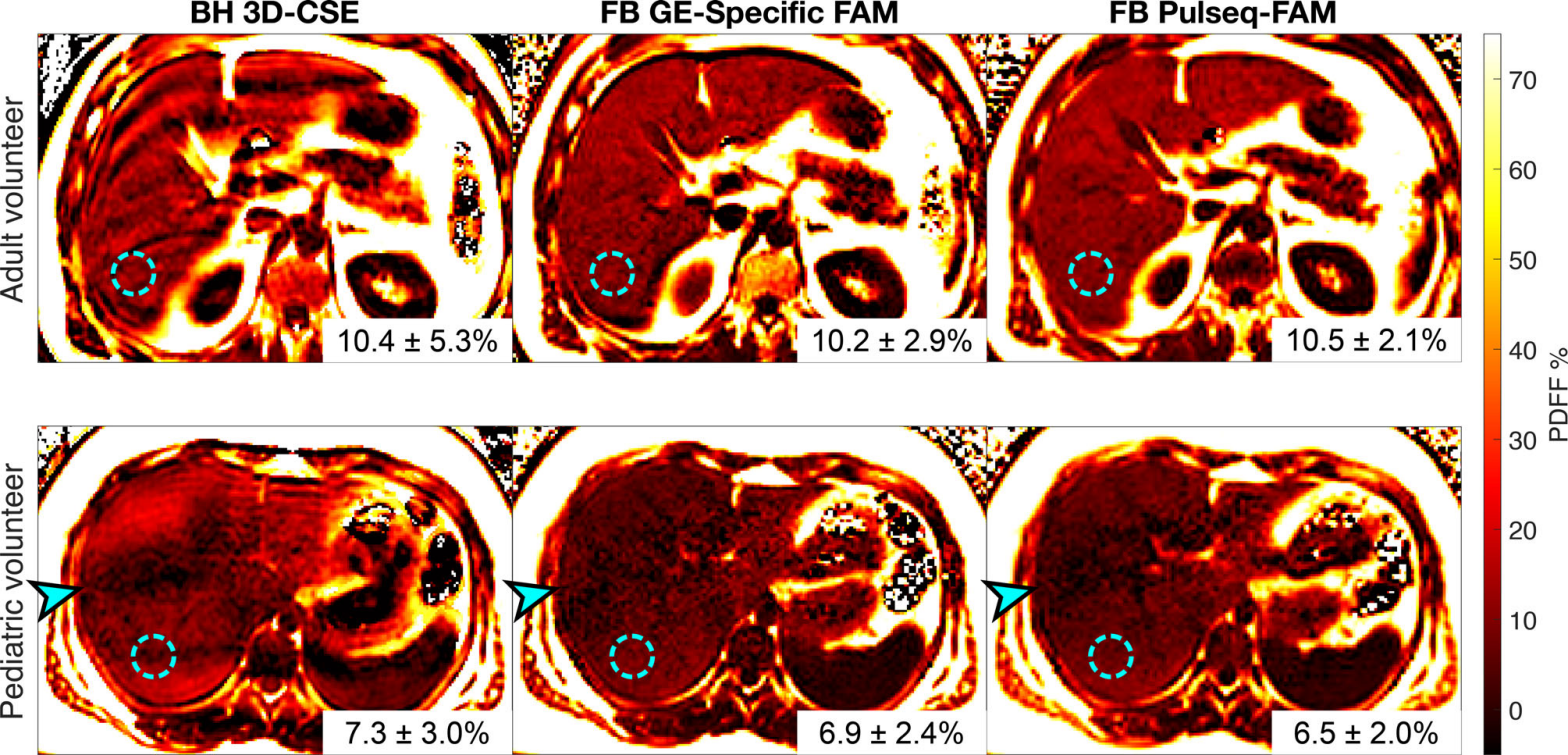
Pulseq‐FAM is accurate with respect to 3D‐CSE and GE‐specific FAM, and shows motion artifact reduction compared to 3D‐CSE. The figure shows an adult patient with suspected steatosis and child with elevated BMI imaged with motion‐sensitive 3D‐CSE in breath‐holding, and motion‐insensitive GE‐specific FAM and Pulseq‐FAM in free‐breathing. 3D‐CSE shows pronounced motion artifacts, likely due to poor breath‐holding compliance. Both GE‐specific FAM and Pulseq‐FAM methods show reduced motion artifacts, even in free‐breathing, and yield PDFF quantification values that agree closely with 3D‐CSE. Note that in the pediatric volunteer, there is likely some real liver PDFF heterogeneity (arrows), as shown in FAM images; although this heterogeneity is also visible in 3D‐CSE, it may be misinterpreted as a motion artifact.

Representative PDFF maps from an adult with suspected steatosis and from a child with elevated BMI are shown in Figure 3. 3D‐CSE PDFF maps show artifactual motion‐induced PDFF inhomogeneity throughout the liver due to poor breath‐holding, whereas GE‐specific FAM and Pulseq‐FAM PDFF maps appear visually similar and free of motion artifacts.

#### 3.3.2 Reader Study

**FIGURE 4:**
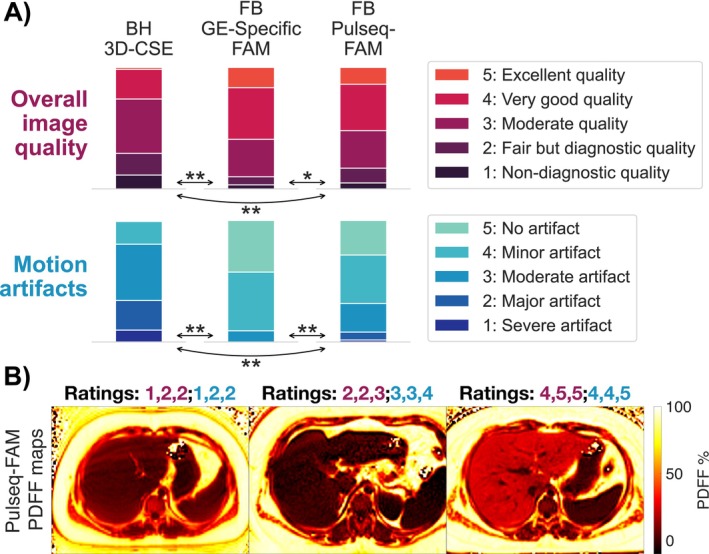
In a reader study, free‐breathing Pulseq‐FAM shows improved image quality and motion artifact reduction compared to breath‐held 3D‐CSE. Shown in (A) are stacked bar plots depicting the distribution of Likert scale ratings in a subset of PDFF maps acquired in adults and children in this study. Two‐tailed Wilcoxon signed‐rank tests were used in paired comparisons to determine significance in differences between ratings for two CSE‐MRI methods. In overall image quality, Pulseq‐FAM showed significant (**p < 0.01) improvement versus 3D‐CSE, but also lower image quality (*p < 0.05) than GE‐specific FAM. In motion artifacts, Pulseq‐FAM also showed significant (*p* < 0.01) improvement versus 3D‐CSE, but also more motion artifacts (*p* < 0.01) than GE‐specific FAM. Shown in (B) are sample Pulseq‐FAM PDFF maps with low, moderate, and high ratings. Semicolons separate overall image quality and motion artifact scores, and commas separate ratings from different raters.

Figure 4 illustrates results of the reader study for the volunteers imaged with the updated Pulseq interpreter. Both free‐breathing GE‐specific FAM and Pulseq‐FAM show improved overall image quality and motion artifacts compared to breath‐held 3D‐CSE (*p* < 0.01). GE‐specific FAM shows superior image quality (*p* < 0.05) and motion artifacts (*p* < 0.01) compared to Pulseq‐FAM. Particularly in children, breath‐held 3D‐CSE performed relatively poorly, with the highest rating received being 3 (moderate quality/motion artifacts) from any of the raters (see Figure S4). In comparison, Pulseq‐FAM received a 4 or 5 motion artifact rating (minor artifact/no artifact) 59% of the time. Figure S5 shows reader study results for pre‐ and post‐interpreter update images, as well as combined results. The updated interpreter shows improved image quality and motion artifacts. Agreement between readers was good, with ICC(2,k) of 0.83 and 0.90 for the overall image quality and motion artifact ratings, respectively.

#### 3.3.3 Quantitative Analysis

**FIGURE 5:**
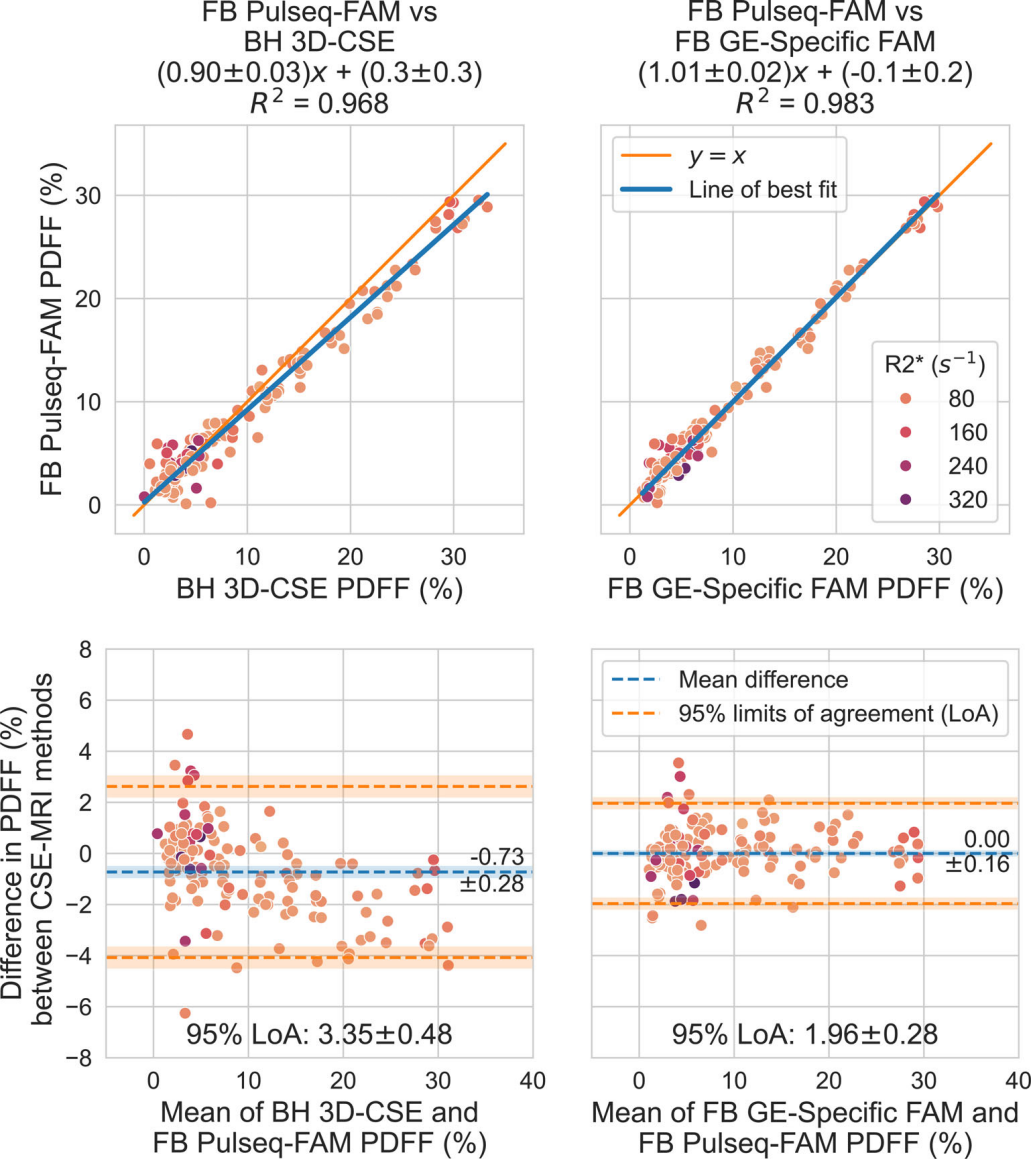
Pulseq‐FAM shows good in vivo accuracy compared to 3D‐CSE and GE‐specific FAM. Shown are linear regressions and Bland–Altman comparisons of whole‐liver PDFF values at 1.5T and 3.0T, averaged between ROIs placed on the nine Couinaud segments of the liver. Pulseq‐FAM shows some underestimation of PDFF at elevated PDFF levels compared to 3D‐CSE, which may be attributable to T1‐related bias in 3D‐CSE. Pulseq‐FAM and GE‐specific FAM show excellent agreement with virtually no bias. Points are colored by R2* as indicated on the color scale in the top right.

Figure 5 shows the strong agreement between Pulseq‐FAM and 3D‐CSE and GE‐specific FAM, in whole‐liver PDFF measurements (95% LoA = 3.4% and 2.0% PDFF, respectively). Pulseq‐FAM shows slight but significant underestimation of PDFF relative to 3D‐CSE (slope = 0.90 ± 0.03) but not GE‐specific FAM (slope = 1.01 ± 0.02). Figure S6 shows that bias results are similar before and after the Pulseq interpreter update.

**FIGURE 6:**
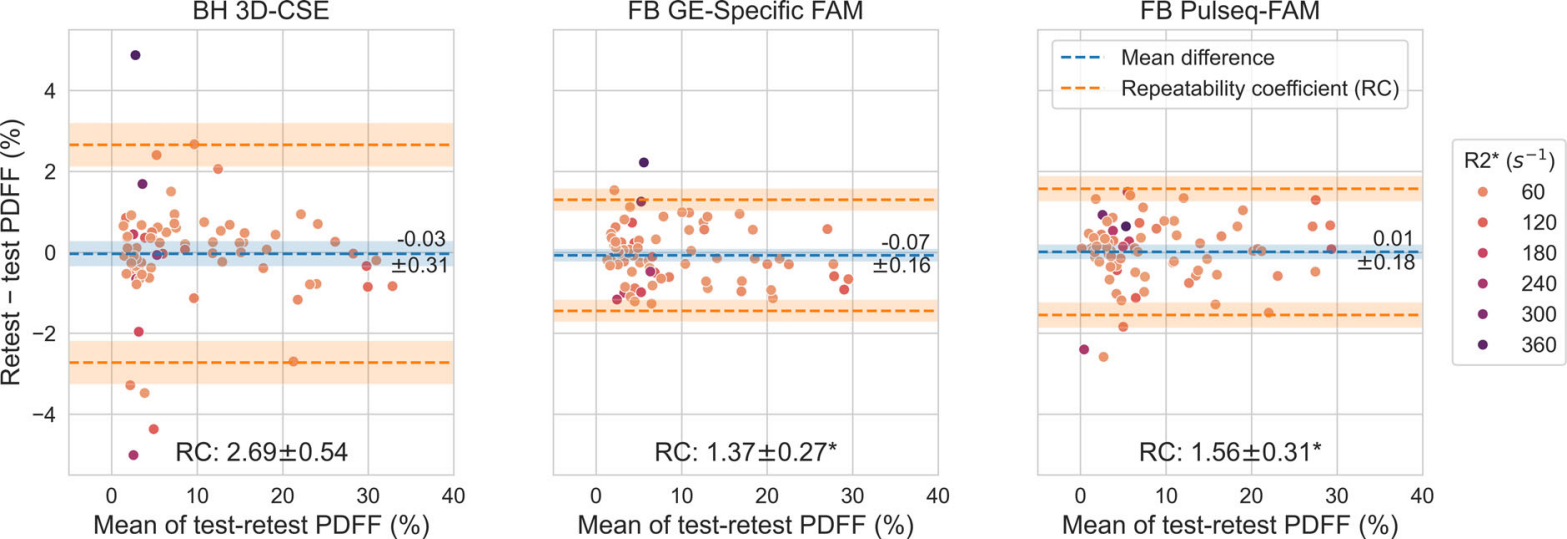
Free‐breathing Pulseq‐FAM shows improved (*p < 0.05) test–retest repeatability in vivo compared to breath‐held 3D‐CSE, and similar repeatability as free‐breathing GE‐specific FAM. Shown are Bland–Altman analyses of repeatability of whole‐liver PDFF values at 1.5T and 3.0T, averaged between ROIs placed on the nine Couinaud segments of the liver. Volunteers were imaged in test–retest after removing them from the bore, repositioning, and repeating localizers. Points are colored by R2* as indicated on the color scale on the right.

Figure 6 shows test–retest repeatability results for the three CSE‐MRI methods in this work. Pulseq‐FAM shows significantly (*p* < 0.05) improved repeatability (repeatability coefficient, RC = 1.6% PDFF) compared to 3D‐CSE (RC = 2.7% PDFF), as well as similar repeatability as GE‐specific FAM (RC = 1.4% PDFF). Figure S7 shows that repeatability results are similar before and after the Pulseq interpreter update.

**FIGURE 7:**
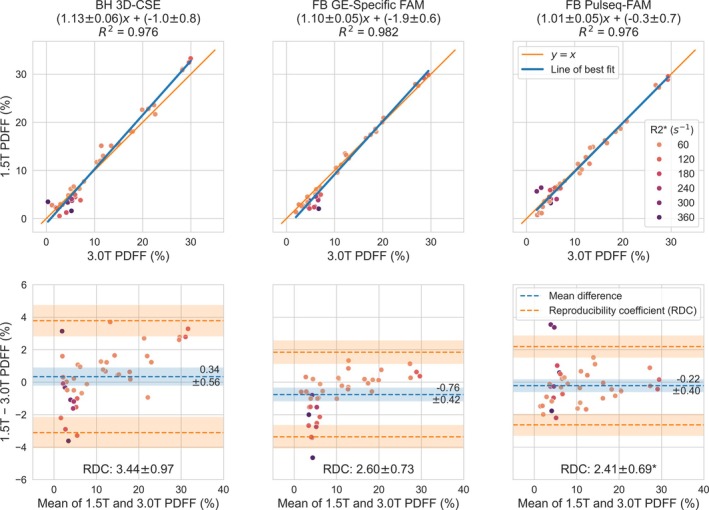
Free‐breathing Pulseq‐FAM shows improved (*p < 0.05) cross‐field‐strength reproducibility in vivo compared to breath‐held 3D‐CSE, and similar reproducibility as free‐breathing GE‐specific FAM. A subset of adult volunteers were imaged at both 1.5T and 3.0T in the same study visit. Shown are Bland–Altman analyses of reproducibility between 1.5T and 3.0T whole‐liver PDFF values, averaged between ROIs placed on the nine Couinaud segments of the liver on the acquisitions at each field strength.

Figure 7 shows between‐field strength reproducibility results for the 21 adult subjects who were imaged at both 1.5T and 3.0T. Pulseq‐FAM shows significantly (*p* < 0.05) improved reproducibility (reproducibility coefficient, RDC = 2.4% PDFF) compared to 3D‐CSE (RDC = 3.4% PDFF), and similar reproducibility as GE‐specific FAM (RDC = 2.6% PDFF). Generally, 3D‐CSE at 1.5T compared to 3.0T appears to slightly underestimate PDFF in cases of high R2*, and overestimate in cases of normal R2*. GE‐specific FAM at 1.5T compared to 3.0T appears to underestimate PDFF in cases of high R2*, while Pulseq‐FAM shows minimal systematic differences across field strengths.

## 4 Discussion

In this work, we successfully developed and validated a vendor‐agnostic, Pulseq‐based method for motion‐insensitive PDFF mapping. Further, we have evaluated the quantitative performance and reproducibility of this method across multiple vendors, field strengths, and patient populations. Pulseq‐FAM is accurate compared to commercially available, validated [15], FDA‐approved 3D‐CSE methods, as well as GE‐specific FAM, which has been validated in research and clinical environments [21, 22, 28, 56].

Interestingly, a small but significant underestimation of PDFF with Pulseq‐FAM, relative to 3D‐CSE in patients with elevated PDFF, was observed. However, it is likely that this relative bias is at least partially attributable to residual T1‐related bias in 3D‐CSE [40, 41]. For example, as used in this work at 3.0T, 3D‐CSE with TR = 6.4 ms and flip angle = 3° would be expected to measure PDFF at 31.7% for a true PDFF of 30%, assuming previously published T1 values [57] for liver fat (382 ms) and water (809 ms). As shown in the phantom study in this work and previous work [22], FAM‐based methods are intrinsically less T1‐biased due to the use of centric encoding.

Further, Pulseq‐FAM shows similar in vivo repeatability and reproducibility advantages as GE‐specific FAM relative to 3D‐CSE. In phantoms, the between‐vendor reproducibility of Pulseq‐FAM is also superior to using each vendor's 3D‐CSE method on their respective systems. Repeatability and between‐field strength reproducibility coefficients for 3D‐CSE measured in this work were similar to those measured in previous work [58].

Technical disadvantages of the Pulseq‐FAM method include worse noise performance in phantoms compared to 3D‐CSE, as expected for a 2D‐encoded method such as Pulseq‐FAM. This difference was greater at 1.5T compared to 3T, which may be partially attributable to the larger flip angles used in 3D‐CSE at 1.5T compared to 3T. Noise performance in phantoms was similar to GE‐specific FAM, which is also 2D‐encoded. In vivo, the noise performance is more than compensated by the lack of motion‐induced variability of the FAM method, leading to superior repeatability in patients.

Pulseq‐FAM was also observed to have greater field strength dependence in PDFF quantification in the phantom study, although Pulseq‐FAM showed excellent reproducibility in vivo across field strengths. One further disadvantage of Pulseq‐FAM is that it showed worsened image quality and motion artifacts compared to GE‐specific FAM, as revealed through the in vivo reader study. These differences may be partly related to RF pulse design, where 3D‐CSE and GE‐specific FAM use proprietary pulses designed by vendors while the current Pulseq‐FAM implementation uses a relatively simple apodized sinc design.

Notably, this implementation broadens the availability of motion‐robust FAM across vendors and field strengths, in particular at 0.55T, since this field strength is available from one vendor only. Considering its quantitative performance, reproducibility, and accessibility across patient groups of varied breath‐holding ability, Pulseq‐FAM may be helpful for clinical trials where PDFF is used as an endpoint to assess treatment response [59, 60], especially in multi‐site trials that use MR systems from multiple vendors. In the longer term, the demonstration of the feasibility of the FAM technique on several vendors' hardware may encourage more vendors to implement FAM in their own software frameworks. This may result in innovations where FAM may be combined with each vendor's proprietary technologies, such as AI‐powered image denoising [61]. At present, vendors generally offer 3D‐CSE methods for fat quantification, which are motion‐sensitive. Such methods may complicate clinical workflows by requiring breath‐holding and requiring re‐imaging in case of inadvertent respiratory motion [20, 21]. Further, breath‐holding may be infeasible for some patient groups. In comparison, the wide dissemination of motion‐insensitive methods such as FAM may reduce workflow complication, reduce rework, improve feasibility across patient groups, and ultimately decrease the cost and increase the value of CSE‐MRI exams.

Multiple other motion‐robust MR‐based PDFF quantification methods have been proposed [17, 26, 27]. Several of these methods make use of non‐Cartesian sampling trajectories to re‐visit the center of k‐space repeatedly to mitigate the effects of motion [62]. In comparison, however, FAM is a modification of a relatively uncomplicated 2D‐encoded, Cartesian, multi‐echo spoiled gradient echo acquisition, leading to relatively straightforward implementation in Pulseq. In particular, due to its Cartesian sampling, FAM is less sensitive to system imperfections such as gradient delays and eddy currents relative to non‐Cartesian methods, which often require system‐specific calibration and/or correction [63, 64]. Indeed, in this work, the only system‐specific step of the post‐acquisition workflow was the loading of k‐space data; all other postprocessing steps, such as Fourier transform, coil combination, and PDFF fitting were identical between systems. The use of Cartesian sampling is a strength that ensures excellent reproducibility, without any system‐specific calibrations, and will help facilitate the widespread dissemination of this method.

There are several limitations to the Pulseq‐FAM method and to our study design. As identified in previous works [22] evaluating GE‐specific FAM, slices in a 2D‐encoded method like FAM may not form a consistent liver volume in free‐breathing. This could be resolved through breath‐holding [65] (including splitting into multiple short breath holds, as is feasible in 2D‐encoded methods), prospective respiratory triggering, or image registration in postprocessing [56, 66]. This implementation of Pulseq‐FAM also does not implement parallel imaging acceleration [22], bipolar readouts [65, 67], or simultaneous multi‐slice (SMS) acceleration [68], as previously demonstrated in combination with 2D‐encoded CSE‐MRI (on a vendor‐specific platform). Implementing these techniques would shorten the temporal footprint of each acquired slice, which may lead to motion artifact reduction, and shorten overall acquisition time. Implementing bipolar readouts would also shorten echo spacing, which would be expected to improve performance in the presence of high R2* [55, 65]; however, system‐specific gradient delay effects and sensitivity to B_0_ shimming imperfections may complicate postprocessing for a cross‐vendor bipolar FAM implementation [67]. Parallel imaging acceleration [69, 70, 71], bipolar readouts [72, 73], and SMS acceleration [74] have all been demonstrated in other Pulseq pulse sequences and may be added to future versions of Pulseq‐FAM.

Other limitations of this work include the single‐site, single‐vendor in vivo validation study. Future work may evaluate Pulseq‐FAM in vivo at a clinical site where multiple vendors' MR systems are available, to enable direct evaluation of in vivo reproducibility. Pulseq‐FAM was also acquired on only one Philips system in the multi‐center phantom study. Future studies may benefit from evaluating Pulseq‐FAM in a set of MR systems more balanced across vendors, as well as including more vendors with emerging or available Pulseq interpreters, such as Canon [75, 76] and United Imaging [77].

In conclusion, we have successfully developed and evaluated a vendor‐agnostic, flip‐angle‐modulated CSE‐MRI method for quantification of PDFF. We demonstrated that this method is an accurate, precise, and motion‐insensitive PDFF quantification method, with excellent test–retest repeatability and reproducibility between vendors, systems, and field strengths. The free‐breathing acquisition and wide availability of Pulseq‐FAM work synergistically to enhance its feasibility across diverse patient populations and imaging centers. Pulseq‐FAM may represent a significant step in the overall accessibility and reproducibility of MR‐based fat quantification.

## Conflicts of Interest

The following authors are affiliated with the University of Wisconsin, which receives research support from GE HealthCare: Alexandra A. Anagnostopoulos, Aaron L. Carrel, Julius F. Heidenreich, Diego Hernando, Lu Mao, Scott B. Reeder, Amirhossein Roshanshad, Felix Schön, Jitka Starekova, Daiki Tamada, and Jiayi Tang. Jiayi Tang is a shareholder of GE HealthCare. Jean H. Brittain, Jeff Kammerman and David Rutkowski are employees of Calimetrix, LLC, and Jean H. Brittain, Diego Hernando and Scott B. Reeder are co‐founders of Calimetrix, LLC, which manufactured and loaned to the authors the phantom used in this study. Eugene Milshteyn is an employee of GE HealthCare. William A. Grissom receives research support from Siemens Healthineers. Yun Jiang receives research support from Siemens Healthineers and Cook Medical. Xiaodong Zhong is affiliated with the University of California, Los Angeles, which receives research support from Siemens Healthineers/Siemens Medical Solutions USA, Inc. Xiaodong Zhong reports relevant conflicts of interest with Siemens that include stocks, funding grants, and travel support. Xiaodong Zhong has patents issued and pending to Siemens that are relevant to the topic of this work.

## Supporting information


**Figure S1:** Flip angles used at each field strength for FAM‐based methods, as determined by numerical optimization. Pulseq‐FAM and GE‐specific FAM use identical flip angles, since the relevant parameters input into the optimization are shared (TR, resolution, assumed fat and water T1).
**Figure S2:** Pulseq‐FAM shows minimal T1‐related bias, regardless of vendor, MR system, and field strength, in a phantom modulated in PDFF and T1. The figure shows example PDFF maps acquired with Pulseq‐FAM, in a phantom with simultaneously controlled combinations of PDFF (0%, 10%, 20%, and 30%) and T1_water_ (T1_w_) (200, 600, 1000, and 1400 ms). Pulseq‐FAM shows accurate and reproducible PDFF measurements in the phantom across all systems in this study. Further, over the wide range of T1_w_ in the phantom, Pulseq‐FAM shows minimal T1‐related bias.
**Figure S3:** Pulseq‐FAM has slightly lower noise performance than 3D‐CSE (**p < 0.01) at 3T, and substantially lower noise performance than 3D‐CSE (*p* < 0.01) at 1.5T. The plot shows voxel‐wise standard deviation (SD) values in ROIs drawn on phantom vials, as a surrogate for noise performance. Because noise may be affected by hardware factors, each system's SD values are normalized to that system's median SD ROI in the 3D‐CSE acquisition. At 3T, Pulseq‐FAM performs only slightly worse in noise performance compared to 3D‐CSE. At 1.5T, Pulseq‐FAM shows worse noise performance than 3D‐CSE. At both field strengths, Pulseq‐FAM and GE‐specific FAM show similar noise performance (*p* = 0.08 and 0.06 at 1.5T and 3T, respectively).
**Figure S4:** Breath‐held 3D‐CSE shows especially poor performance in children, and free‐breathing Pulseq‐FAM improves image quality and motion artifacts. Shown are reader study results post‐interpreter update, broken down by age cohort (adults with suspected liver steatosis or iron overload, and children with normal or elevated BMI). 3D‐CSE shows relatively poor image quality in children, with no raters giving a score over 3 (moderate image quality/motion artifacts) for any 3D‐CSE acquisitions. Pulseq‐FAM improves image quality and motion artifacts compared to 3D‐CSE, but also performs worse than GE‐specific FAM (*p < 0.05 and **p < 0.01).
**Figure S5:** A Pulseq interpreter update for GE systems appears to improve image quality. Shown are reader study results pre‐ and post‐Pulseq interpreter update, as well as combined results. The interpreter update improved RF scaling accuracy, which may have resulted in flip angles closer to those designed through the FAM formalism during the acquisition. This may improve image quality by creating filtering effects closer to that intended in the FAM method.
**Figure S6:** A Pulseq interpreter update for GE systems appears to have little effect on the bias of Pulseq‐FAM relative to 3D‐CSE and GE‐specific FAM. Shown are linear regression results between Pulseq‐FAM and 3D‐CSE or GE‐specific FAM, pre‐ and post‐Pulseq interpreter update. The interpreter update improved RF scaling accuracy, which may change image filtering and appearance. However, quantitative results are likely to be robust in a fairly wide range of reasonable filtering effects, as identified in previous work.
**Figure S7:** A Pulseq interpreter update for GE systems appears to have little effect on in vivo repeatability for Pulseq‐FAM. Shown are Bland–Altman repeatability analyses for whole‐liver average PDFF values, pre‐ and post‐Pulseq interpreter update. Volunteers were imaged in test–retest after removing them from the bore, repositioning, and repeating localizers. The interpreter update improved RF scaling accuracy, which may change image filtering and appearance. However, quantitative results are likely to be robust in a fairly wide range of reasonable filtering effects, as identified in previous work.

## Data Availability

A repository [78] is available for download at https://zenodo.org/records/16989988. Linear mixed‐effects models, underlying data, and code for permutation testing for significance of differences in parameters between models are included. Also included are Matlab scripts for generating the Pulseq sequences used in the work, as well as pre‐compiled sequences ready for execution on MR systems with a Pulseq interpreter. This work also made use of the ISMRM fat‐water fitting toolbox described by Hu et al. [46], and toolbox access is available through the procedure described in that work.
