# Supplementary material for: Highly Reproducible, Vendor‐Agnostic, Motion‐Insensitive Liver PDFF Mapping at 0.55T, 1.5T, and 3T
Source: Magn Reson Med. 2025 Dec 12;95(5):2797–813. doi: 10.1002/mrm.70223 (PMC12962202; doi:10.1002/mrm.70223)
Supplement: Supplementary file 1 — Figure S1: Flip angles used at each field strength for FAM‐based methods, as determined by numerical optimization. Pulseq‐FAM and GE‐specific FAM use identical flip angles, since the relevant parameters input into the optimization are shared (TR, resolution, assumed fat and water T1). Figure S2: Pulseq‐FAM shows minimal T1‐related bias, regardless of vendor, MR system, and field strength, in a phantom modulated in PDFF and T1. The figure shows example PDFF maps acquired with Pulseq‐FAM, in a phantom with simultaneously controlled combinations of PDFF (0%, 10%, 20%, and 30%) and T1water (T1w) (200, 600, 1000, and 1400 ms). Pulseq‐FAM shows accurate and reproducible PDFF measurements in the phantom across all systems in this study. Further, over the wide range of T1w in the phantom, Pulseq‐FAM shows minimal T1‐related bias. Figure S3: Pulseq‐FAM has slightly lower noise performance than 3D‐CSE (**p < 0.01) at 3T, and substantially lower noise performance than 3D‐CSE (p < 0.01) at 1.5T. The plot shows voxel‐wise standard deviation (SD) values in ROIs drawn on phantom vials, as a surrogate for noise performance. Because noise may be affected by hardware factors, each system's SD values are normalized to that system's median SD ROI in the 3D‐CSE acquisition. At 3T, Pulseq‐FAM performs only slightly worse in noise performance compared to 3D‐CSE. At 1.5T, Pulseq‐FAM shows worse noise performance than 3D‐CSE. At both field strengths, Pulseq‐FAM and GE‐specific FAM show similar noise performance (p = 0.08 and 0.06 at 1.5T and 3T, respectively). Figure S4: Breath‐held 3D‐CSE shows especially poor performance in children, and free‐breathing Pulseq‐FAM improves image quality and motion artifacts. Shown are reader study results post‐interpreter update, broken down by age cohort (adults with suspected liver steatosis or iron overload, and children with normal or elevated BMI). 3D‐CSE shows relatively poor image quality in children, with no raters giving a score over 3 (moderate i [file MRM-95-2797-s001.docx]

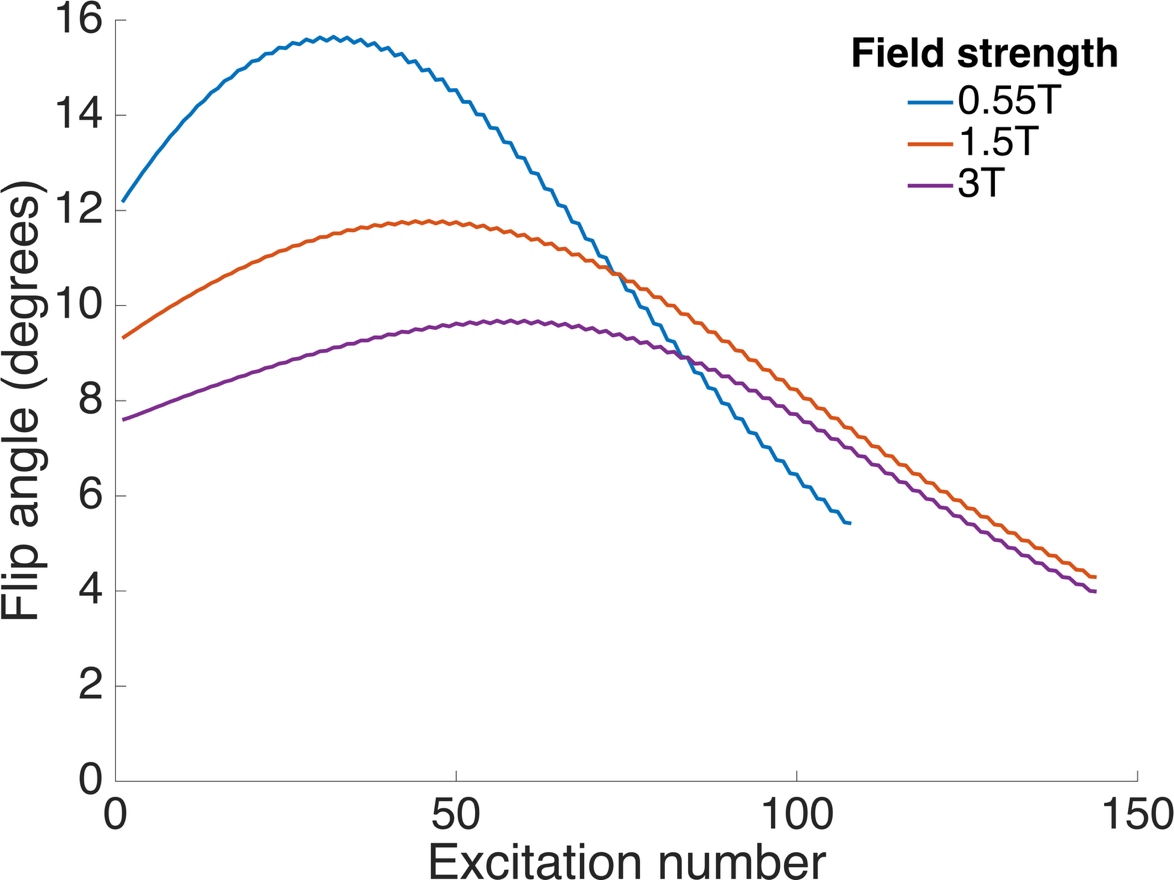


**Figure S1:** Flip angles used at each field strength for FAM-based methods, as determined by numerical optimization. Pulseq-FAM and GE-specific FAM use identical flip angles, since the relevant parameters input into the optimization are shared (TR, resolution, assumed fat and water T1.)


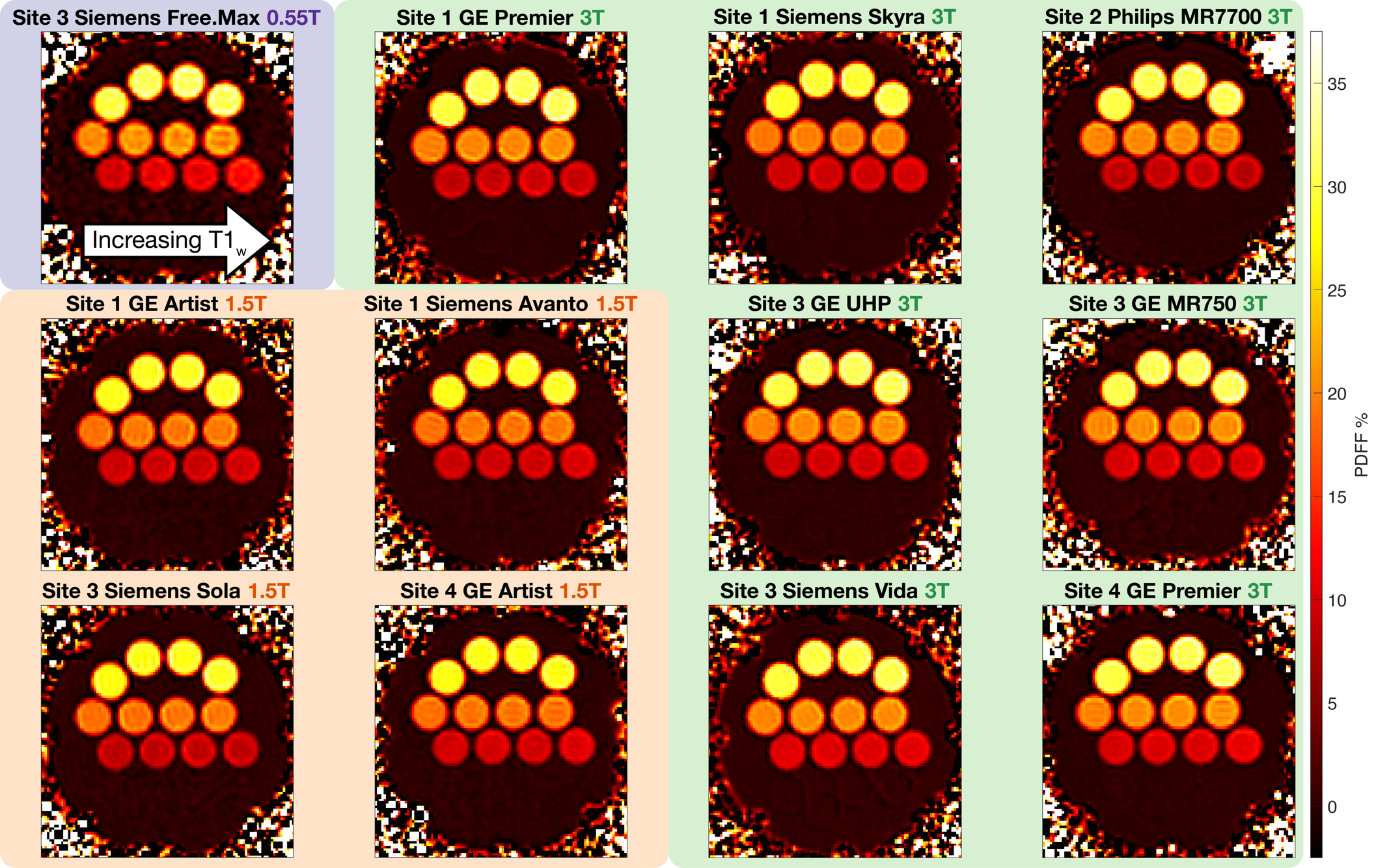


**Figure S2:** Pulseq-FAM shows minimal T1-related bias, regardless of vendor, MR system, and field strength, in a phantom modulated in PDFF and T1. The figure shows example PDFF maps acquired with Pulseq-FAM, in a phantom with simultaneously controlled combinations of PDFF (0, 10, 20, 30%) and T1_water_ (T1_w_) (200, 600, 1000, 1400 ms). Pulseq-FAM shows accurate and reproducible PDFF measurements in the phantom across all systems in this study. Further, over the wide range of T1_w_ in the phantom, Pulseq-FAM shows minimal T1-related bias.


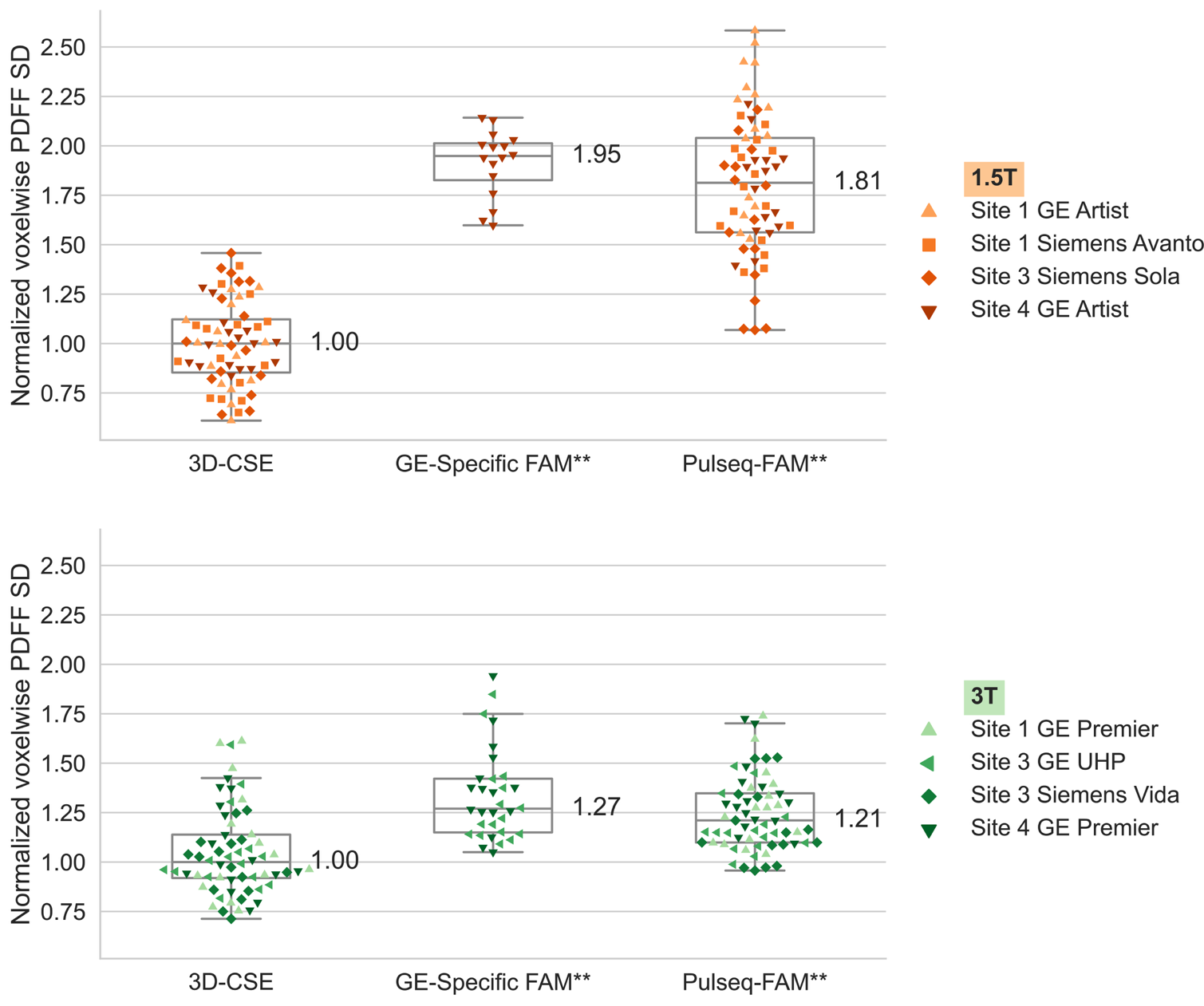


**Figure S3:** Pulseq-FAM has slightly lower noise performance than 3D-CSE (** = *p*<0.01) at 3T, and substantially lower noise performance than 3D-CSE (*p*<0.01) at 1.5T. The plot shows voxel-wise standard deviation (SD) values in ROIs drawn on phantom vials, as a surrogate for noise performance. Because noise may be affected by hardware factors, each system’s SD values are normalized to that system’s median SD ROI in the 3D-CSE acquisition. At 3T, Pulseq-FAM performs only slightly worse in noise performance compared to 3D-CSE. At 1.5T, Pulseq-FAM shows worse noise performance than 3D-CSE. At both field strengths, Pulseq-FAM and GE-specific FAM show similar noise performance (*p*=0.08 and 0.06 at 1.5T and 3T respectively).


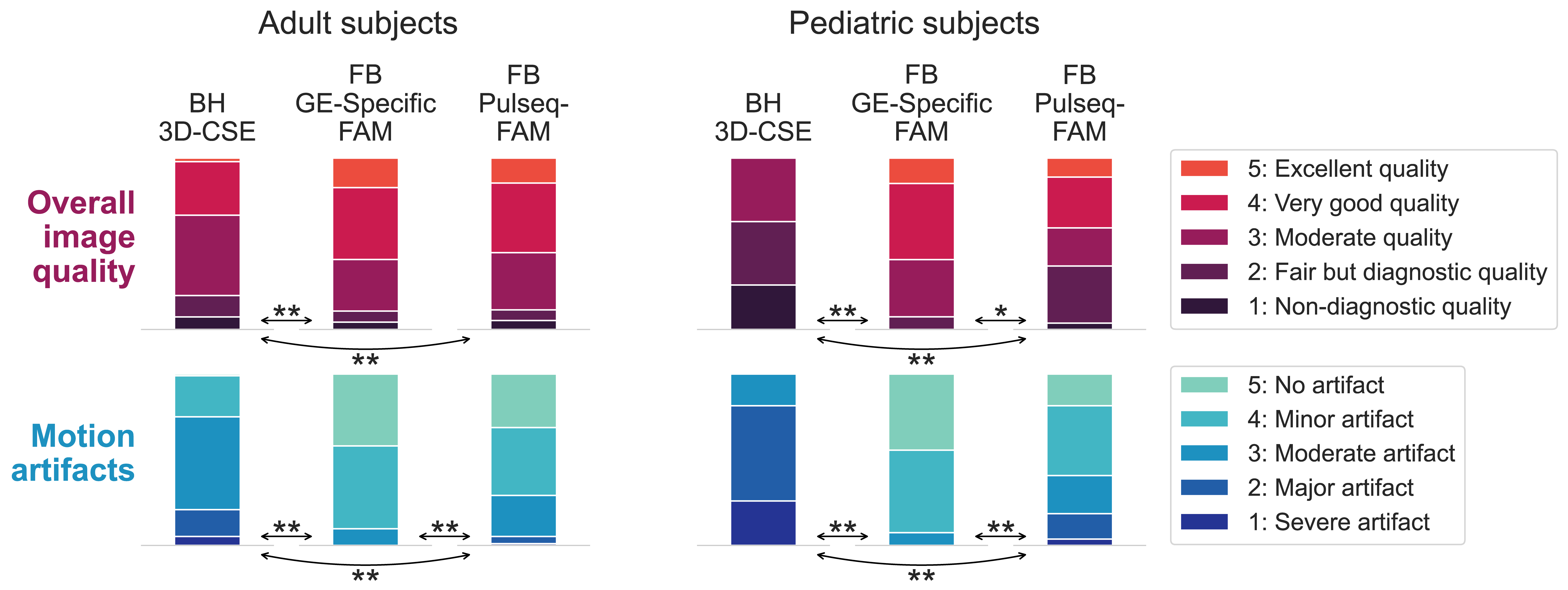


**Figure S4:** Breath-held 3D-CSE shows especially poor performance in children, and free-breathing Pulseq-FAM improves image quality and motion artifacts. Shown are reader study results post-interpreter update, broken down by age cohort (adults with suspected liver steatosis or iron overload, and children with normal or elevated BMI.) 3D-CSE shows relatively poor image quality in children, with no raters giving a score over 3 (moderate image quality/motion artifacts) for any 3D-CSE acquisitions. Pulseq-FAM improves image quality and motion artifacts compared to 3D-CSE, but also performs worse than GE-specific FAM (* = *p*<0.05 and ** = *p*<0.01).


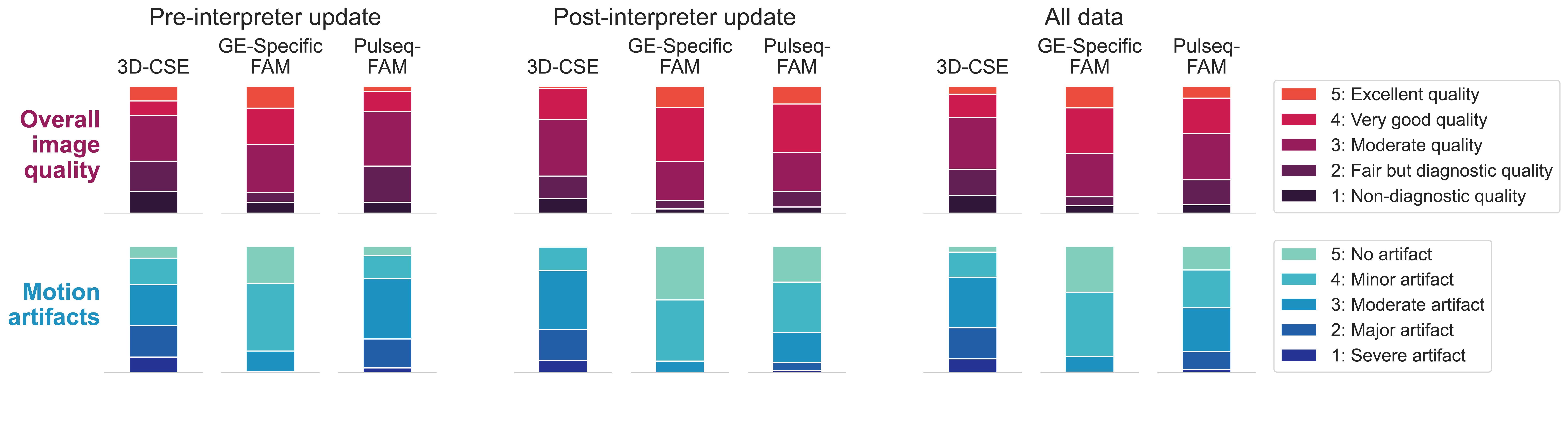


**Figure S5:** A Pulseq interpreter update for GE systems appears to improve image quality. Shown are reader study results pre- and post-Pulseq interpreter update, as well as combined results. The interpreter update improved RF scaling accuracy, which may have resulted in flip angles closer to those designed through the FAM formalism during the acquisition. This may improve image quality by creating filtering effects closer to that intended in the FAM method.


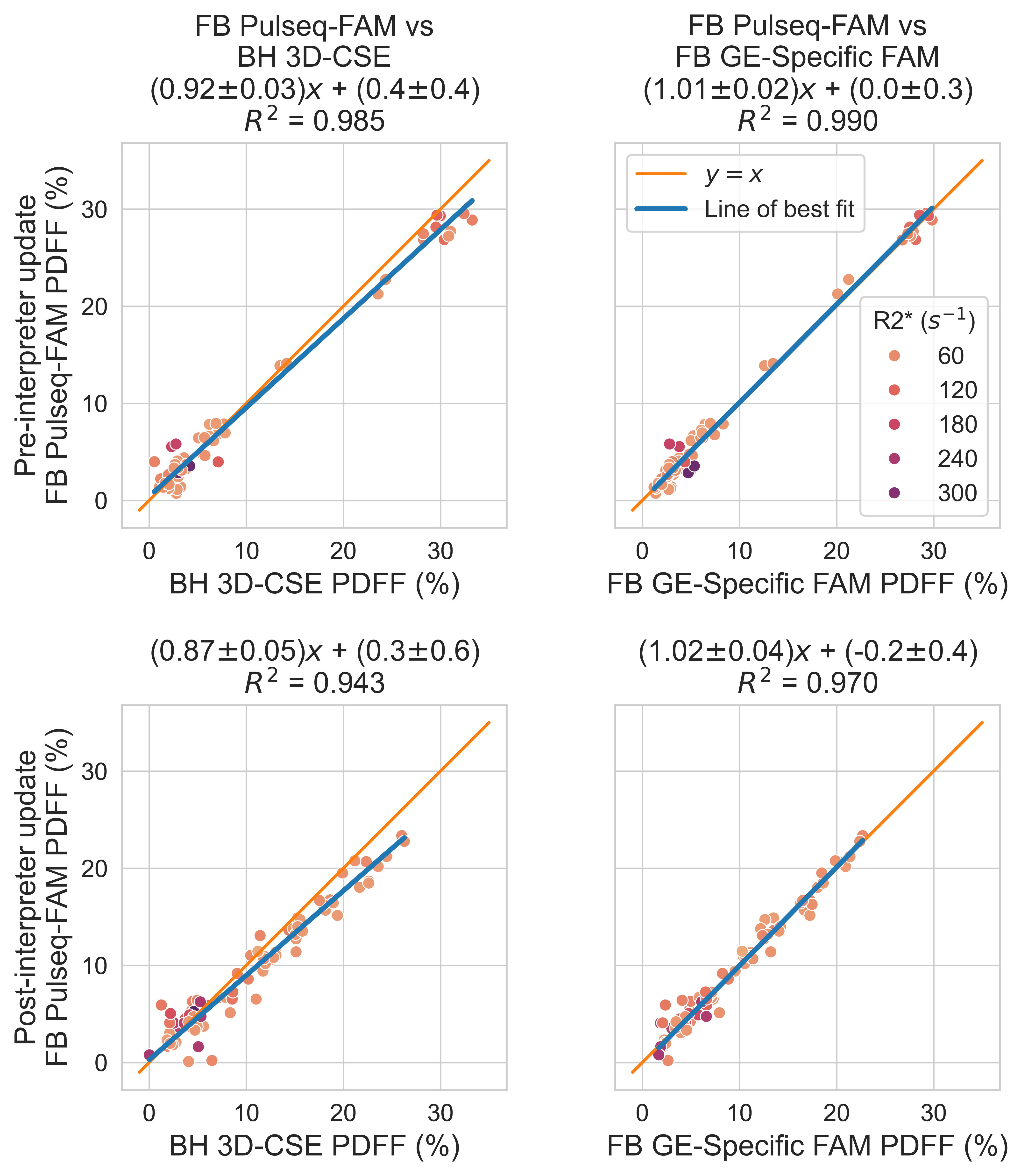


**Figure S6:** A Pulseq interpreter update for GE systems appears to have little effect on the bias of Pulseq-FAM relative to 3D-CSE and GE-specific FAM. Shown are linear regression results between Pulseq-FAM and 3D-CSE or GE-specific FAM, pre- and post-Pulseq interpreter update. The interpreter update improved RF scaling accuracy, which may change image filtering and appearance. However, quantitative results are likely to be robust in a fairly wide range of reasonable filtering effects, as identified in previous work.


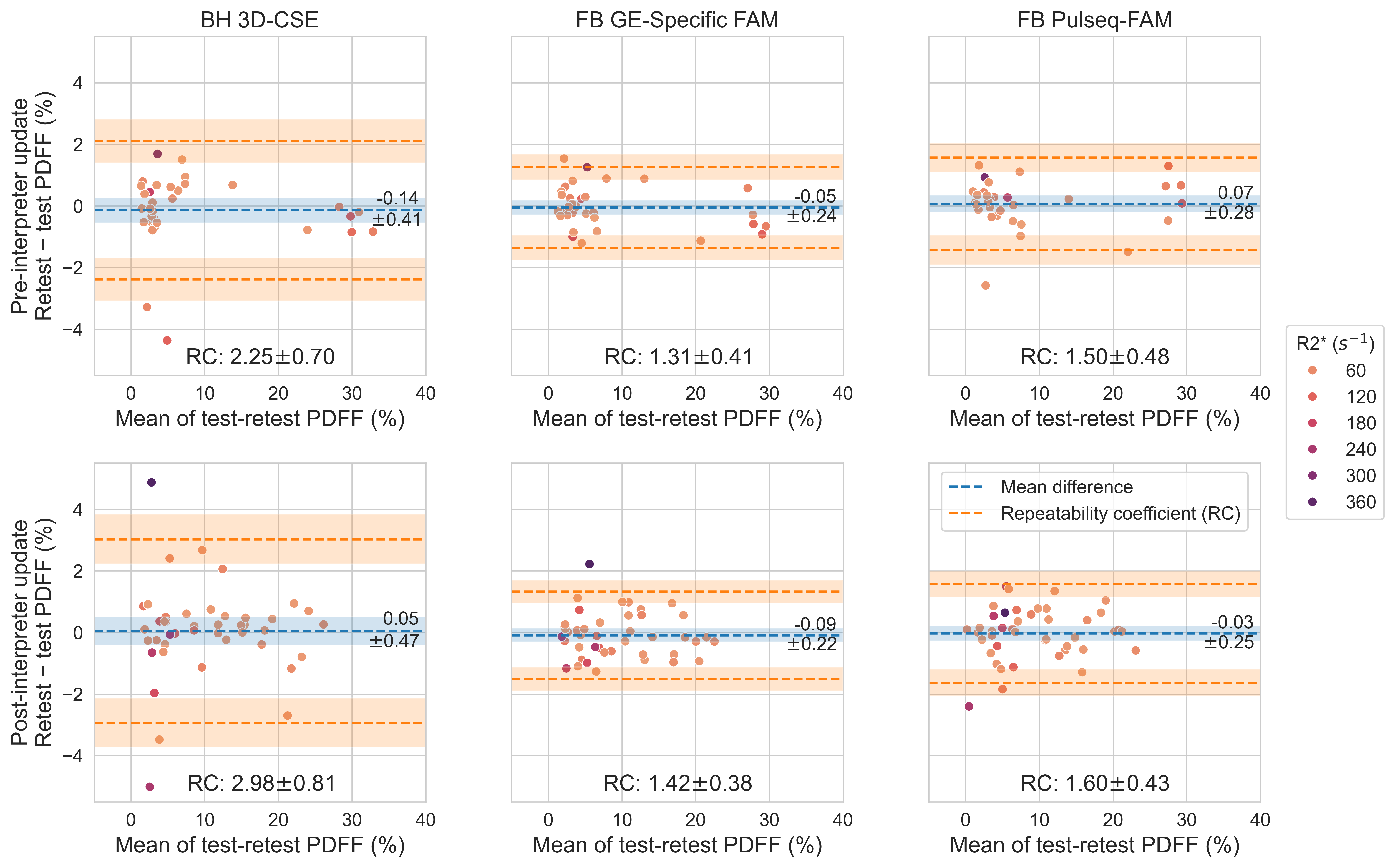


**Figure S7:** A Pulseq interpreter update for GE systems appears to have little effect on in vivo repeatability for Pulseq-FAM. Shown are Bland-Altman repeatability analyses for whole-liver average PDFF values, pre- and post-Pulseq interpreter update. Volunteers were imaged in test-retest after removing them from the bore, repositioning, and repeating localizers. The interpreter update improved RF scaling accuracy, which may change image filtering and appearance. However, quantitative results are likely to be robust in a fairly wide range of reasonable filtering effects, as identified in previous work.
